# Cracks propagation characteristics of double-hole delay blasting in soft-hard composite rock mass

**DOI:** 10.1038/s41598-023-35748-7

**Published:** 2023-05-30

**Authors:** Jianbin Cui, Liangfu Xie, Yongjun Qin, Xuejun Liu, Jianhu Wang, Jiangu Qian

**Affiliations:** 1 College of Civil Engineering and Architecture, Xinjiang University, Urumqi, 830017 China; 2 Xinjiang Civil Engineering Technology Research Center, Urumqi, 830017 China; 3 Xinjiang Academy of Architectural Science (Limited Liability Company), Urumqi, 83002 China; 4 College of Civil Engineering, Tongji University, Shanghai, 200092 China

**Keywords:** Civil engineering, Petrology

## Abstract

By researching the distance between blasthole and interface of soft-hard rock strata, as well as the time of delay detonation, blasting effect of the rock mass will be more controllable. Firstly, validity of numerical method was authenticated from three angles: blasting coupled stress field, ratio of crushing zone radius to blasthole radius, and crack network state. Under the condition of soft-hard rock strata, numerical model of double-hole blasting was established by using PFC^2D^. Then delay blasting experiments were carried out under different relative positions of blasthole and interface. Ultimately, results were analyzed from three perspectives: crack network, crack quantity and rock fragment. Results show that: (1) When detonated in hard rock, if between interface and blasthole distance is greater than twice crushing zone radius, the closer blasthole is to the interface, the more obvious the “hook” phenomenon between the two blastholes is. With increasing delayed initiation time, “hook” phenomenon will weaken or even disappear. (2) Based on the crack information initiated in hard rock, the law of crack number varying with thickness of hard rock and delay time is obtained. (3) For initiation in hard rock, crack extension range is large, but less fragments are formed. The law is opposite to that initiation in structural plane and soft rock. Fragmentation area increases exponentially with increasing soft rock thickness, and exponential function is obtained.

## Introduction

Blasting technology is frequently used in mine slope^1,2^, underground mining^3,4^ and other geological projects. In engineering, double-hole initiation or group-hole initiation is usually adopted. Hence, interaction of blastholes is always inevitable. Nowadays, the research on double-hole initiation or group-hole initiation chiefly focuses on distance of blastholes^5^, in-situ stress^6^, delay time^7^ and charging mode^8^. By comparing the model test results of single-hole and double-hole initiation, Yang et al.^9^ believed that, at the midpoint of two blastholes, the peak strain in double-hole blasting is 2.4–2.7 times that of single-hole blasting, and the stress superposition effect of double-hole blasting is much greater than 2. Pu et al.^10^ explored the impact of drilling distance as well as lagging time on double-hole initiation based on numerical simulation. It was found that the increase of hole distance was detrimental to the crack combination and crack length at the common pilot hole. Wu et al.^11^ used LS-DYNA numerical simulation analysis to compare and study cracks propagation of double-hole detonation and penetration phenomenon of cracks in elliptical bipolar linear charging explosion and general explosion, which found that the former seriously influences directional crack’s formation. Chen et al.^12^ studied fracturing characteristics under coupling action of static pressure and delay blasting based on the concrete double-hole explosion test, and found that fragmentation degree after detonation is tightly associated with the two factors. Zhang et al.^13^ used the ANASYS/LS-DYNA coupling method to simulate the two hole blasting of slit charge under two-way equal pressure and two-way different pressure under high ground stress. It was found that under the condition of two-way equal pressure, the greater the in-situ stress, the greater the suppression of blasting effect. Under two-way unequal pressure, vertical cracks will be severely inhibited by in-situ stress, and cracks after detonation is easier to extend in high stress direction.

The study on double-hole initiation chiefly focuses on mentioned fields, and the majority of study subjects are homogeneous lithologic rock mass, but in practical engineering, natural rock mass, with plenty of structural planes such as joints, fractures and fault fracture zones, are often encountered^14–18^. In natural rock mass, soft and hard composite strata are quite common. For instance, in Xinjiang, China, coal mines usually have interbedding, alternative and soft-hard rock strata^19^. In tunneling, special geological conditions of soft-hard rock strata are more common at the heading face^20,21^. Interface between soft rock and hard rock is similar to structural plane such as joint fissure, which also severely influences propagation of stress wave. Different from joint fissure, stress wave can transmit through the interface and continue to propagate. In addition, comparing with hard rock, failure mode and crack pattern of soft rock is greatly different^22^, so the blasting exercise of single lithologic rock mass can not be fully applicable to soft and hard composite rock stratum.

In order to provide guidance for practical engineering, under the condition of soft-hard rock strata, double-hole blasting model was established based on PFC^2D^. The influence of two factors on blasting effect, which are the relative position between interface and blasthole as well as delayed initiation time, are further studied. Ultimately, blasting results are analyzed from three angles. The conclusions are helpful to control the blasting effect and can supply guidance for actual blasting engineering.

## Theoretical analysis for blasting in deep rock masses

**Figure 1:**
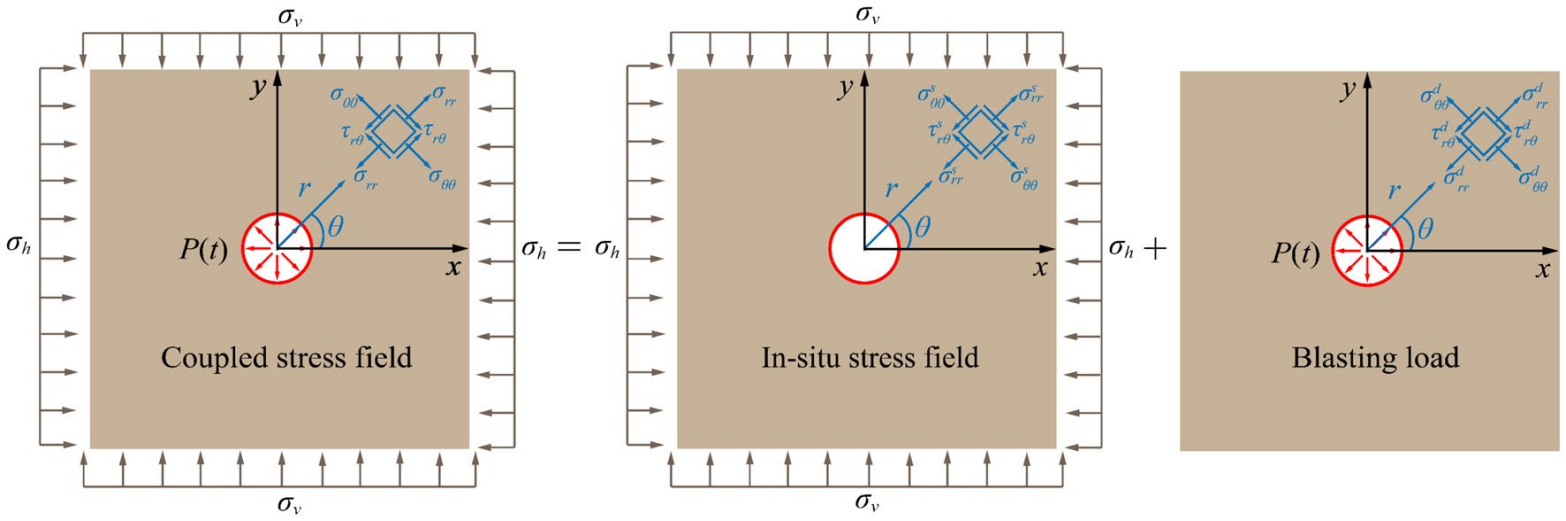
Coupled stress field of blasting in deep rock mass^23^.

As shown in Fig. 1, the schematic diagram of elastodynamic problem under plane strain problem. In an elastic homogeneous medium of infinite length and width, a round hole is existing whose radius is *a*. At the same time, there will be horizontal stress *σ*_h_ and vertical stress *σ*_v_.

Dynamic load *P*(*t*) caused by explosion is exerted to the borehole wall and further diffuses into the rock medium. Therefore, considering the influence of buried depth, stress state in the process of rock mass blasting can be superimposed by static in-situ stress field and dynamic stress field which is induced by blasting load.

Therefore, the analytical solution of the coupled stress field is expressed as superposition of static stress field^24^ and dynamic stress field^25,26^. And the expression is as follows:

1 $$\left\{ {\begin{array}{*{20}c} \begin{aligned} \sigma_{rr} & = \sigma_{rr}^{s} + \sigma_{rr}^{d} \, \\ \, & { = }\frac{1}{2}(\sigma_{h} + \sigma_{v} )\left( {1 - \frac{{a^{2} }}{{r^{2} }}} \right) + \frac{1}{2}(\sigma_{h} - \sigma_{v} )\left( {1 - \frac{{a^{2} }}{{r^{2} }}} \right)\left( {1 - \frac{{3a^{2} }}{{r^{2} }}} \right)\cos 2\theta + P\frac{r}{a}^{{\left( {2 \pm \frac{0.8\mu }{{1 - 0.8\mu }}} \right)}} \\ \end{aligned} \\ \begin{aligned} \sigma_{\theta \theta } & = \sigma_{\theta \theta }^{s} + \sigma_{\theta \theta }^{d} \, \\ \, & { = }\frac{1}{2}(\sigma_{h} + \sigma_{v} )\left( {1 + \frac{{a^{2} }}{{r^{2} }}} \right) - \frac{1}{2}(\sigma_{h} - \sigma_{v} )\left( {1 + \frac{{3a^{2} }}{{r^{2} }}} \right)\cos 2\theta - \frac{0.8\mu }{{1 - 0.8\mu }}P\frac{r}{a}^{{\left( {2 \pm \frac{0.8\mu }{{1 - 0.8\mu }}} \right)}} \\ \end{aligned} \\ \end{array} } \right.$$

where $\sigma_{rr}^{s}$ and $\sigma_{\theta \theta }^{s}$, in static stress field, are radial stress and hoop stress, respectively; *r* is the distance from blasthole (*r* ≥ *a*); *a* is blasthole’s radius; $\sigma_{rr}^{d}$ and $\sigma_{\theta \theta }^{d}$, in dynamic stress field, are radial stress and hoop stress, respectively; *P* is borehole pressure; $\mu$ is static Poisson's ratio.

## Numerical simulation method of PFC^2D^

### Material constitutive model selection

**Figure 2:**
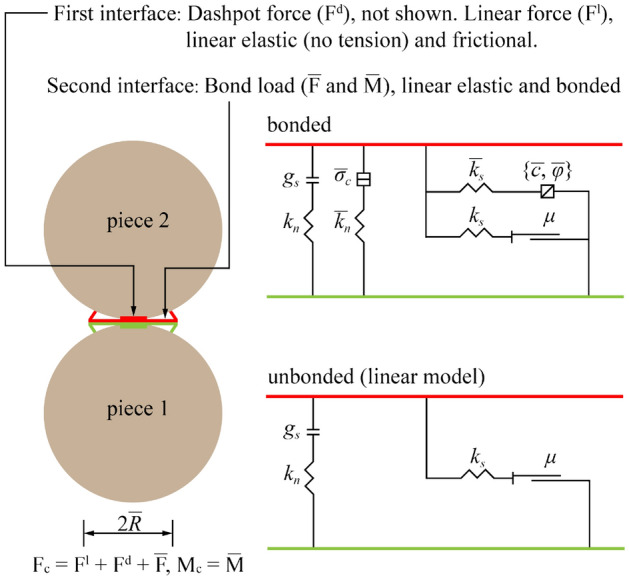
Behavior and rheological components of the linear Parallel Bond Model^29^.

As a discrete element method software to simulate and analyze the deformation and failure characteristics of granular materials, Particle Flow Code (PFC^2D^) is widely used in geotechnical engineering. Especially, it can simulate materials such as rock mass or soil mass. Among the constitutive models in PFC^2D^, Parallel Bond Model (PBM) provides the behavior of two interfaces (Fig. 2): one is the linear elastic interface with infinitely small bearing capacity (no-tension), and the other is the friction interface with finite bearing capacity and moment. BPM is good at simulating the mechanical behavior of rock materials because it can transmit force and moment^25,27,28^. Therefore, PBM is selected to simulate hard rock as well as soft rock.

As shown in Fig. 2, the first interface (linear elastic interface) of PBM is equivalent to linear model, which can not resist relative rotation and adjust sliding by applying Coulomb limit to shear force. When the second interface (friction interface) is bonded, it can resist relative rotation, and its behavior is linear elastic. When the load on the second interface exceeds the strength limit, the bond breaks and does not bear any load. The unbonded parallel bond model is equivalent to the linear model.

In BPM, the maximum values of both tension stress and shear stress are usually the conditions of material failure, which can be expressed as^29^:

2 $$\left\{ {\begin{array}{*{20}l} {\sigma_{t\max } = \frac{{\overline{{F_{n} }} }}{A} + \frac{{\left| {\overline{M} } \right|}}{I}\overline{R} } \hfill \\ {\tau_{\max } = \frac{{\overline{{F_{S} }} }}{A}} \hfill \\ \end{array} } \right.$$

where $\sigma_{t\max }$ and $\tau_{\max }$ are the maximum stress of tension and the maximum stress of shear, respectively; $\overline{{F_{n} }}$ and $\overline{{F_{S} }}$ are normal force and shear force, respectively; *I* and *A* are inertial moment and area of contact cross section, respectively; $\overline{R}$ is radius of contact.

If tension stress is greater than tension strength ($\sigma_{t\max }$ > $\sigma$), contact between the particles will be broken and a new tension failure crack will be generated. Similarly, if shear stress is greater than shear strength ($\tau_{\max }$ > $\tau$), the contact will also be broken and a new shear failure crack will be generated.

### Application of blasting method in PFC^2D^

Particle expansion algorithm^30^ is adopted to realize numerical simulation of blasting process. Under the condition of cylindrical charge, stress wave propagates outward from explosion point, which is expressed as^30^:

3 $$P(t){ = }\frac{{P_{m} }}{2}\left[ {1 - \cos \left( {\frac{{2{\uppi }}}{\Delta T}t} \right)} \right]$$

where $P(t)$ represents initiation pressure; $P_{m}$ represents peak pressure in blasthole whose value is 4 GPa; △*T* is action time of half sinusoidal, which is 10 ms; *t* is time of duration whose value is 20 ms.

The borehole pressure of uncoupled charge *P* will decay rapidly which is^30^:

4 $$P = \frac{1}{8}\rho_{0} D^{2} \left( {\frac{{V_{c} }}{{V_{b} }}} \right)^{3} n$$

where $V_{{\text{c}}}$ is explosive volume; $V_{{\text{b}}}$ is blasthole volume; *n* is factor of increase, whose value range is 8–11.

**Figure 3:**
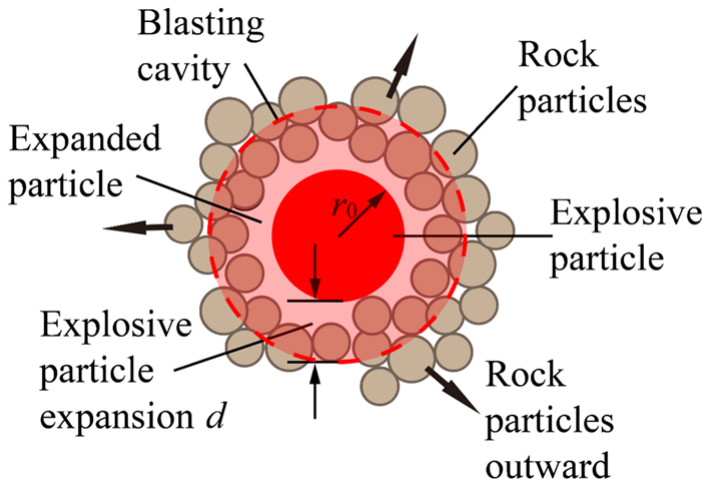
Schematic diagram of particle expansion algorithm^28^.

As shown in Fig. 3, the red particle in the middle is the expansive particle. The sub transparent red circle indicates the expanded particle which can overlap with borehole particles. In this paper, radius of charge is *r*_0_. If the charge enlarges to blasting cavity, pressure of borehole will be *p*. So, thrust force of radial *F* is applied to borehole wall^30^:

5 $$F = K_{{\text{n}}} d = 2{\uppi }r_{{0}} p$$

Particle radius after expansion is^30^:

6 $$d = \frac{{2{\uppi }r_{{0}} p}}{{K_{{\text{n}}} }}$$

7 $$K_{{\text{n}}} { = }\frac{{2(r_{\max } + r_{\min } ){\uppi }p}}{{(r_{\max } - r_{\min } )}}$$

where *K*_n_ represents contact stiffness; *d* represents charge radius after expansion; *r*_max_ and *r*_min_ represent the maximum radius and minimum radius, respectively.

### Numerical model boundary setting

If stress wave reflection occurs at the boundary of numerical model, blasting effect will be seriously affected. Therefore, this paper adopts the boundary considering the dispersion effect proposed by kouroussis et al.^31^ as well as Shi et al.^30^ to deal with the numerical model boundary.

The boundary force can be expressed as^30^:

8 $$F_{b} { = }\left\{ {\begin{array}{*{20}c} { - \xi \, \cdot \, 2\rho C_{{\text{P}}} \dot{u}_{{\text{n}}} r} \\ { - \eta \, \cdot \, 2\rho C_{{\text{s}}} \dot{u}_{{\text{s}}} r} \\ \end{array} } \right.$$

where *ζ* and *η* represent correction coefficients of dispersion effect of longitudinal wave and transverse wave respectively; *C*_P_ and *C*_S_ represent velocity of longitudinal wave and transverse wave, respectively; $\dot{u}_{{\text{n}}}$ and $\dot{u}_{{\text{s}}}$ represent normal velocity of particles and tangential velocity of particles, respectively; *r* represents radius of particle; *ρ* represents density of rock.

## Establishment of blasting numerical model

### Numerical model of single-hole blasting in sandstone

**Figure 4:**
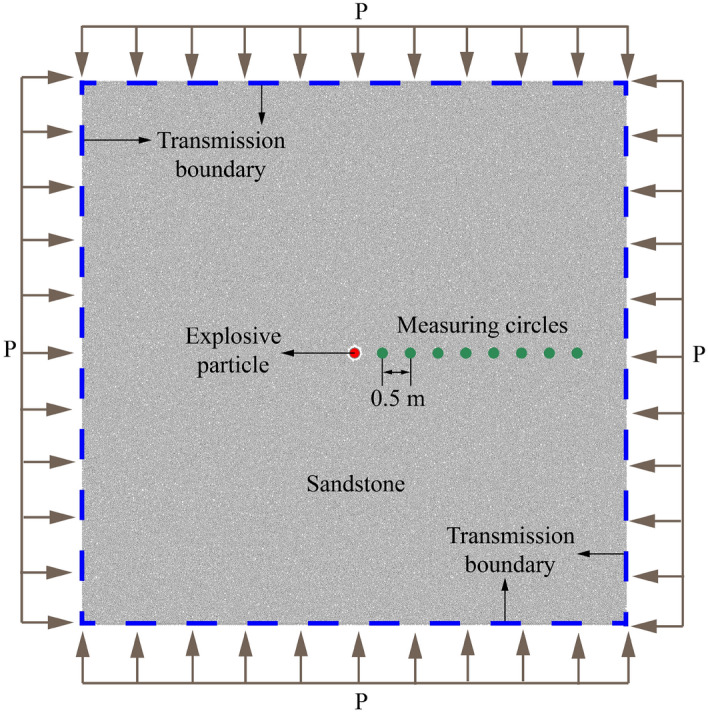
Discrete element method model of single-hole sandstone blasting^33^.

Numerical model of single-hole blasting is built through utilizing PFC^2D^ (Fig. 4), the size of which is 10 m in length and width. The charge particle associates with 10 cm in radius is generated in model center. Based on the method of particle expansion, other particles are created with original radius range 5–7.5 mm. Sandstone is a kind of typical hard rock which is adopted as the research target in numerical blasting experiments of Wei Yuan et al.^29^. They also calibrated the microscopic parameters of sandstone (Table 1). In order to improve the reasonableness of particle expansion algorithm, the same in-situ stress environment as Wei Yuan et al.^29^ is applied. Four boundaries of the model are subjected to the compression pressure of P (P is 5 MPa), and application method of compression pressure is adopted, which is suggested by Cundall et al.^32^.

**Table 1:** Calibration parameters of sandstone^29^.

Linear group	Parallel-bond group
Effective modulus = 51.0 GPa	Bond effective modulus = 42.0 GPa
Friction coefficient = 1.0	Bond stiffness ratio = 1.0
Stiffness ratio = 1.0	Bond tensile strength = 30.0 MPa
	Bond cohesion = 350.0 MPa
	Bond friction = 65°

**Figure 5:**
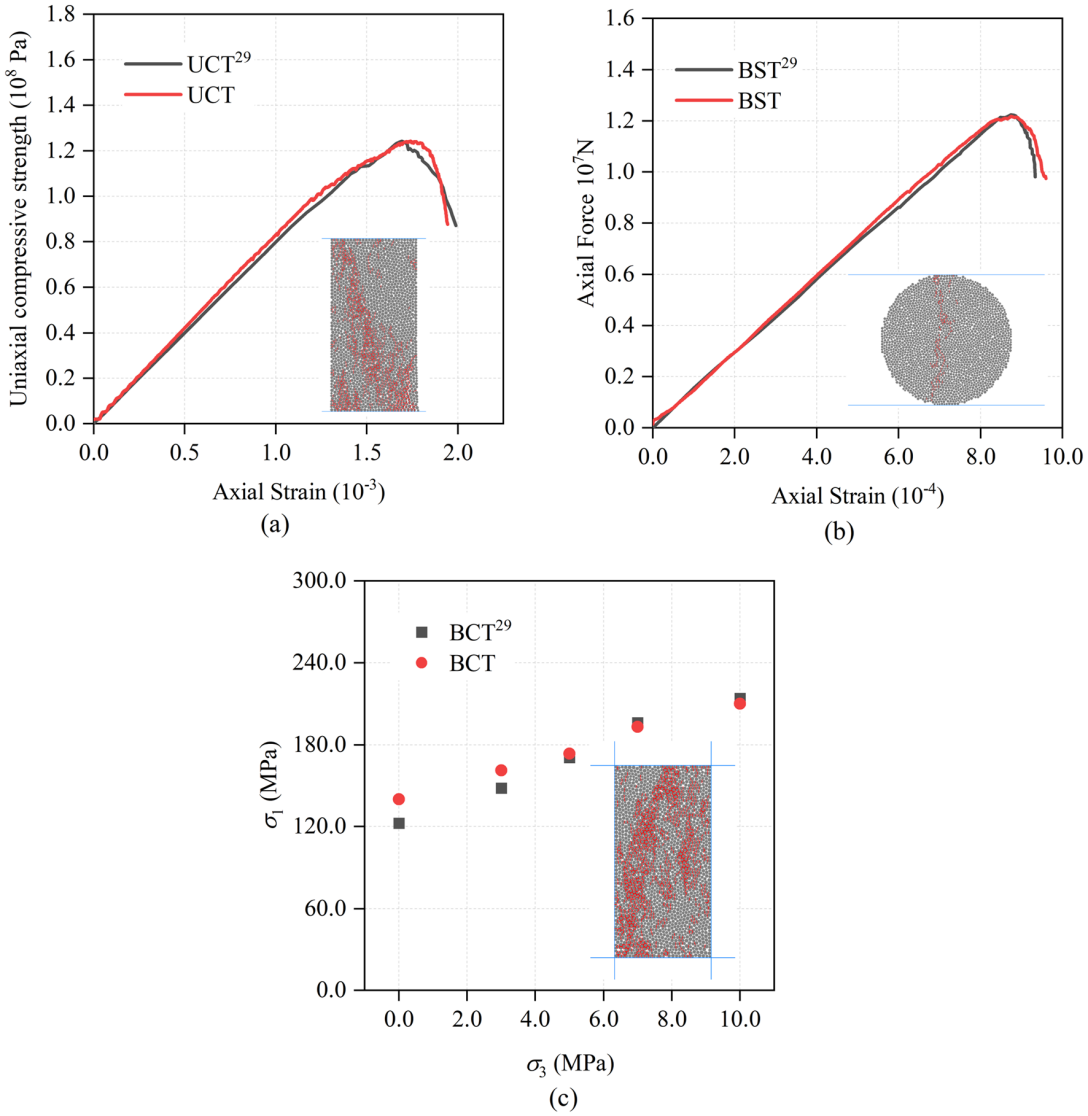
Simulated PFC^2D^ failure of sandstone during Uniaxial Compression Test (UCT) (**a**), Brazilian Splitting Test (BST) (**b**) and Biaxial Compression Test (BCT) (**c**) (red lines indicate cracks)^34^.

So as to prove the reasonableness of microscopic parameters (Table 1), Uniaxial Compression Test (UCT), Biaxial Compression Test (BCT) and Brazilian Splitting Test (BST) are conducted. Rock samples of the UCT and BCT were 50 mm × 100 mm, and the diameter of BST sample is 0.9 m. The range of particle radius, contact constitutive model and particle generation method of the rock samples in the three tests are identical to single-hole sandstone blasting model. The results of rock mechanical parameter test are shown in Fig. 5.

### Validation of numerical blasting method

The rationality of blasting results also needs to verify from multiple angles. Firstly, the coupled stress field after theoretical derivation is compared with the monitoring results during blasting. Then, the ratio of the crushing zone radius to the blasthole radius also can be verified. Finally, the crack network state is compared.

#### Verification of the coupled stress field

In the Eq. (1), both *σ*_*h*_ and *σ*_*v*_ are 5 MPa (gravity is not considered). Blasthole’s radius is 0.143 m. Poisson's ratio of sandstone is 0.188. Charge’s parameters in PFC^2D^ are demonstrated in Table 2. In addition, the stress at corresponding position is monitored by setting measurement circles (Fig. 4) which associated with 10 cm in radius. And the distance between measurement circles is 0.5 m, as well as the distance between blasthole and the nearest measurement circle.

**Table 2:** Main technical indicators of explosive^33^.

Parameters	Density (kg/m^3^)	Radius of the cartridge (cm)	Detonation velocity (m/s)	Radius of the blasthole (cm)
Value	1000	10	3000	14.3

**Figure 6:**
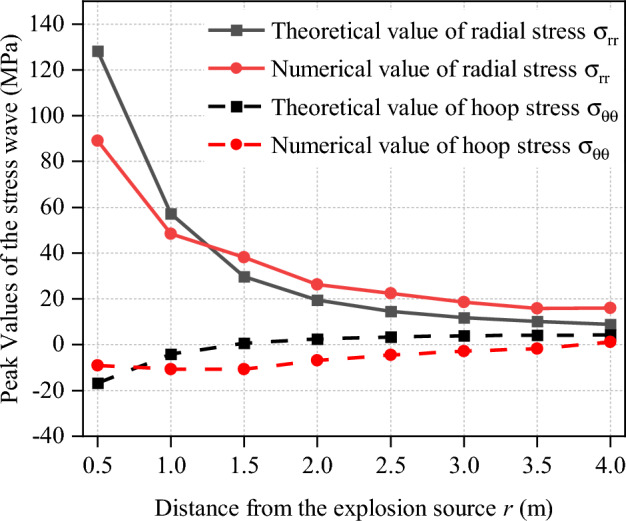
Verification of peak stress of coupled stress field^33^.

After the blasting experiment, the stress at corresponding positions can be read by measurement circles. As shown in Fig. 6, the reasonableness of the blasting method can be proved by contrast with analytical solutions of the stress with the experimental values.

#### Verification of the crushing zone

After blasting, the ratio of the crushing zone radius to the blasthole radius usually follows a certain law, and many scholars evaluate the reasonableness of numerical blasting method through comparing this ratio^6,35^. Hagan^36^ believed that the ratio of the crushed zone thickness relative to the blast cavity diameter is about 5. Hustrulid suggested that most investigators hold the view that the ratio of the crushed zone radius relative to the blasthole radius does not exceed 3–5 blasthole radii^37^. In addition, Kanchibotla et al.^38^ believes that there is a correlation between crushing zone radius and blasthole radius, which can be expressed as:

9 $$r_{c} = r_{0} \sqrt {\frac{{P_{d} }}{{\sigma_{c} }}}$$

where $r_{0}$ is the borehole radius, $P_{d}$ is the detonation pressure and $\sigma_{c}$ is the unconfined compressive strength of the rock.

According to the experimental conditions in this paper and under the condition that the horizontal stress *σ*_*h*_ and the vertical stress *σ*_*v*_ are both 0 MPa, gravity is not considered, $r_{c} /r_{0}$ is calculated to be 5.7. Therefore, by judging the ratio of the crushing zone radius to the blasthole radius, the reasonableness of numerical blasting method is also verified.

#### Verification of the crack network state

**Figure 7:**
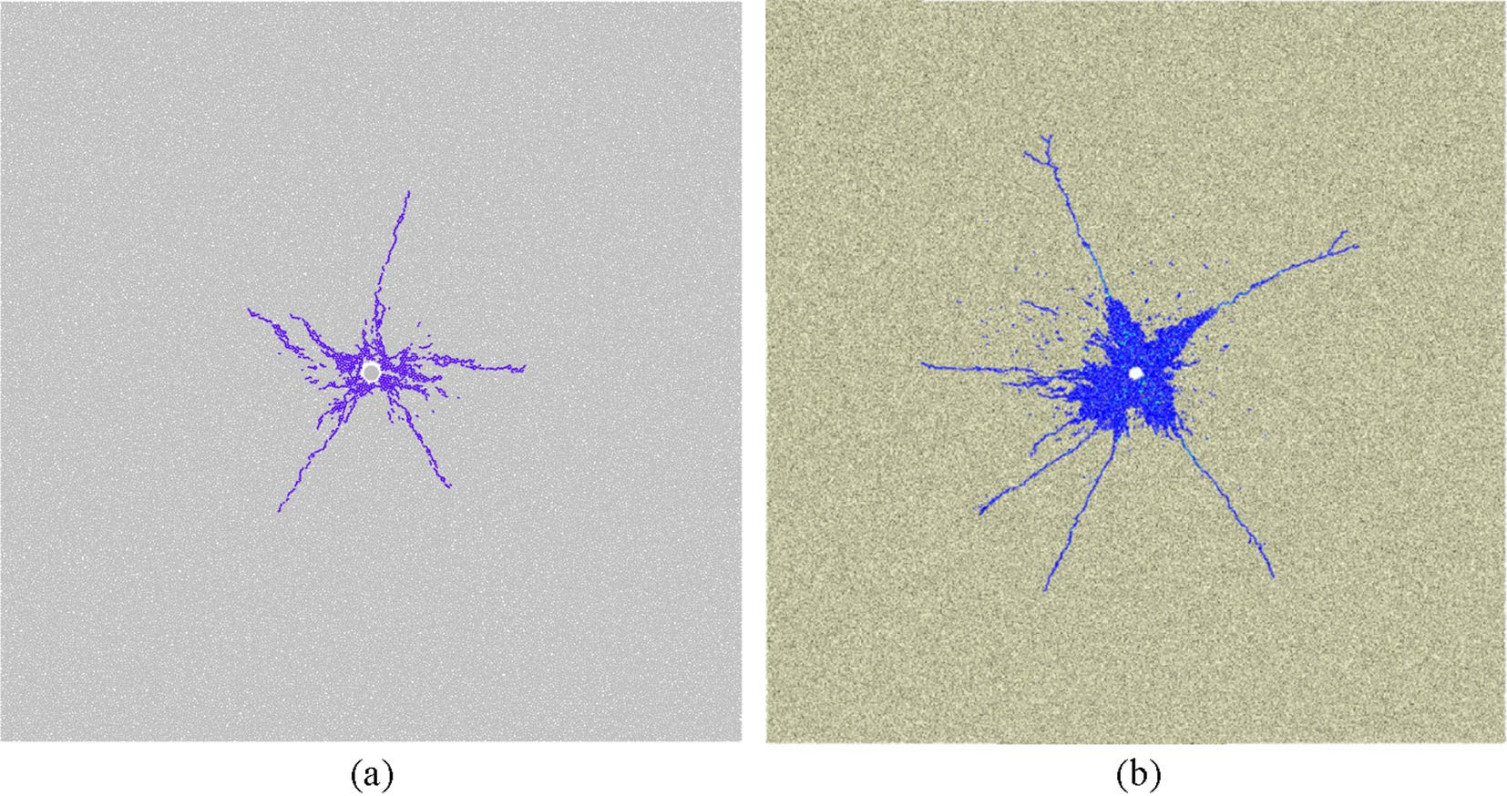
The simulation results (**a**)^34^ in this paper are compared with the result of Wei Yuan et al. (**b**)^29^.

Under the condition of both *σ*_*h*_ and *σ*_*v*_ are 5 MPa (gravity is considered), crack network state of single-hole sandstone obtained in this paper is shown in Fig. 7a. Under the same size of model, micro parameters of sandstone and compression pressure, crack network state is nearly identical with Yuan et al.’s result^29^ (Fig. 7b). Hence, the particle expansion algorithm is authenticated by crack network state.

## Experimental cases setting

**Figure 8:**
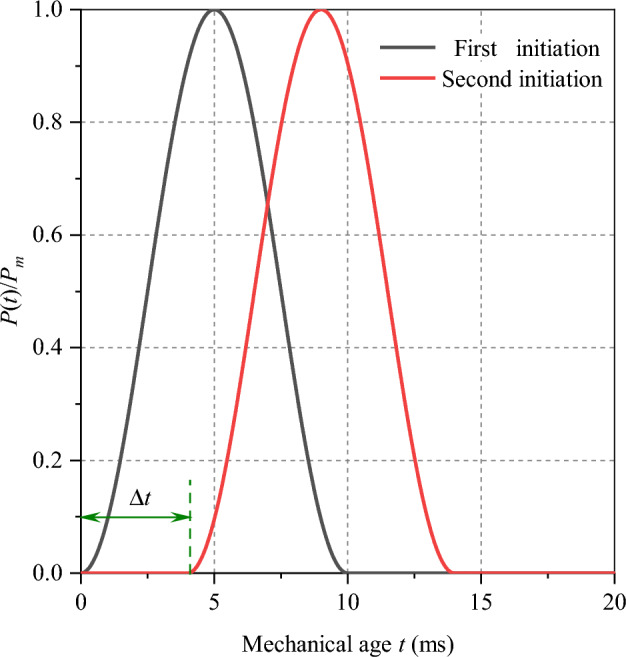
Pressure load variation curve under double-hole blasting.

**Figure 9:**
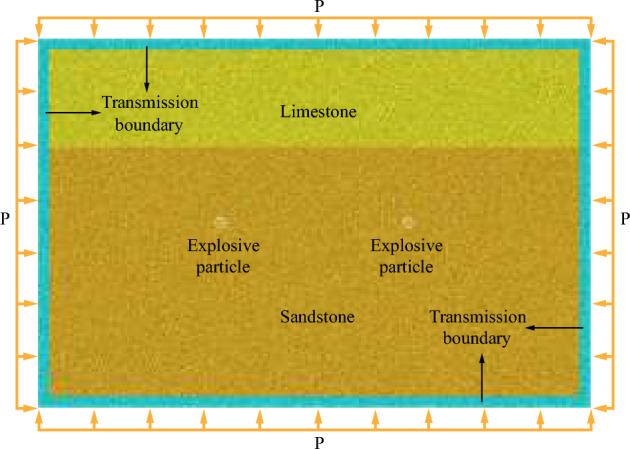
Double-hole blasting numerical model of composite strata^33^.

**Figure 10:**
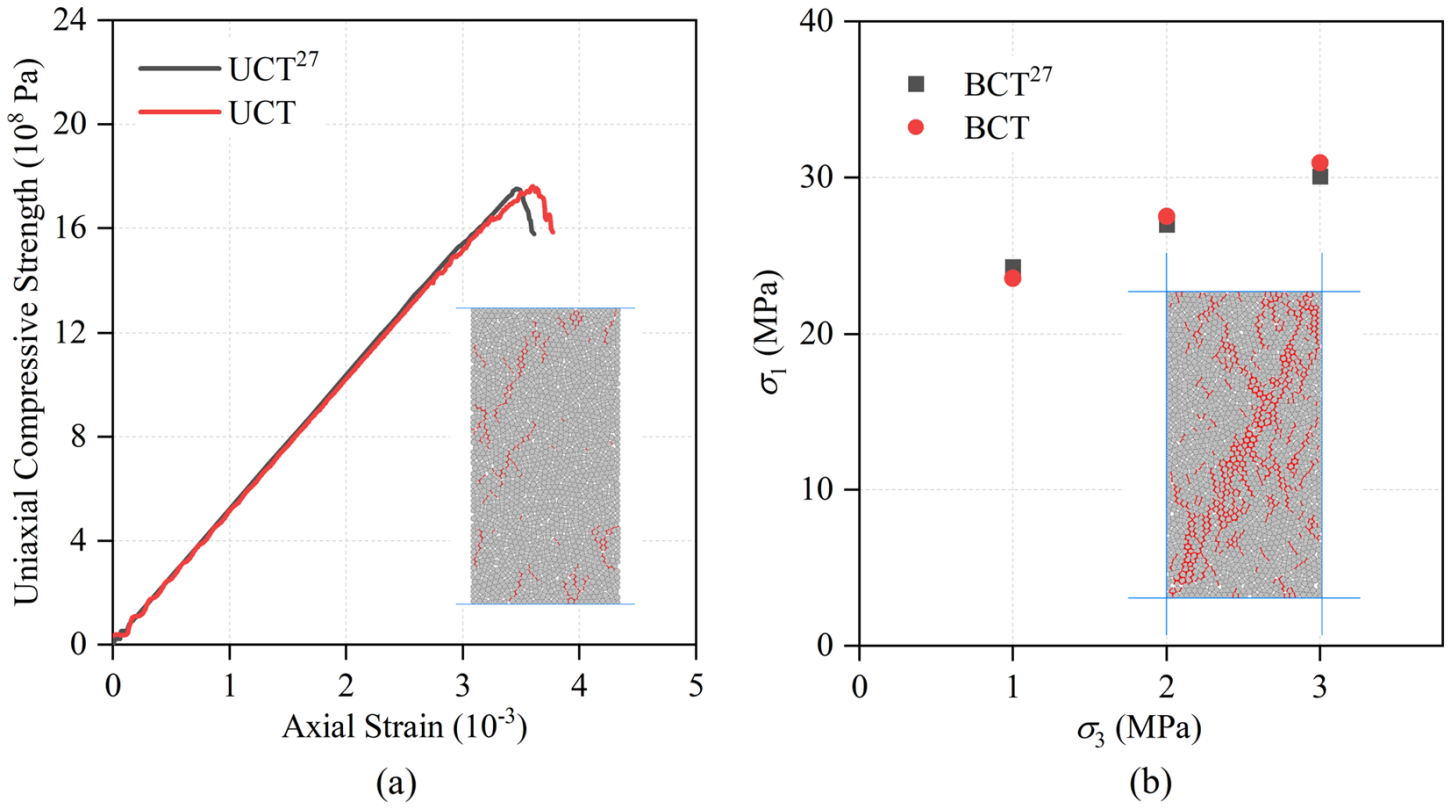
Simulated PFC^2D^ failure of limestone during Uniaxial Compression Test (UCT) (**a**) and Biaxial Compression Test (BCT) (**b**) (red lines indicate cracks)^39^.

Two blastholes of the double-hole blasting model are controlled by two blasting pressure load curves respectively, and the blasting load is applied separately. As shown in Fig. 8, double-hole delayed blasting is by controlling the starting time difference Δ*t* of two blasting pressure load history curves. And then double-hole blasting experiments are carried out under different delay times. Compression pressure, which is 5 MPa, is identical with single-hole blasting model. What’s more, the size of model is 15 m × 10 m. At the same time, the soft rock is introduced into the model (Fig. 9) to research the effect of different thickness of both soft rock and hard rock, and delayed initiation time on blasting effect of rock mass. As shown in Fig. 9, under the delayed initiation cases, left blasthole detonates earlier than right blasthole. By utilizing PFC^2D^, Xie et al.^27^ conducted UCT and BCT, and obtained micro parameters of limestone (Table 3) which was used in this paper. What’s more, UCT and BCT are also conducted with sample size of 50 mm × 100 mm. The results are displayed in the Fig. 10, which proves that micro parameters (Table 3) is accurate.

**Table 3:** Calibration parameters of limestone^27^.

Linear group	Parallel-bond group
Effective modulus = 2.5 GPa	Bond effective modulus = 2.5 GPa
Friction coefficient = 0.2	Bond stiffness ratio = 1.8
Stiffness ratio = 1.8	Bond tensile strength = 10.0 MPa
	Bond cohesion = 5.0 MPa
	Bond friction = 10°

The delay time and distribution cases of two kinds of rocks are shown in Table 4.

**Table 4:**
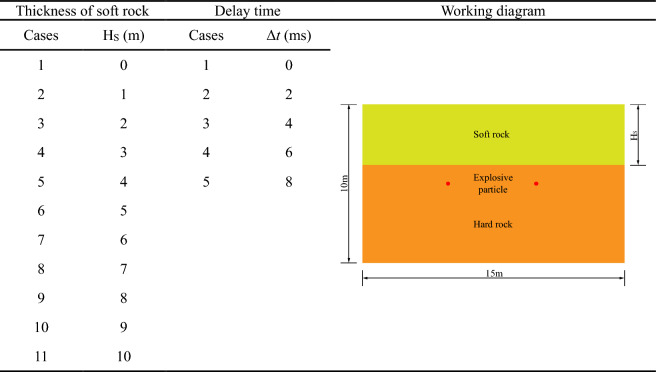
Cases of stratum thickness and delay time^33^.

## Analysis of blasting results

### Crack network

**Figure 11:**
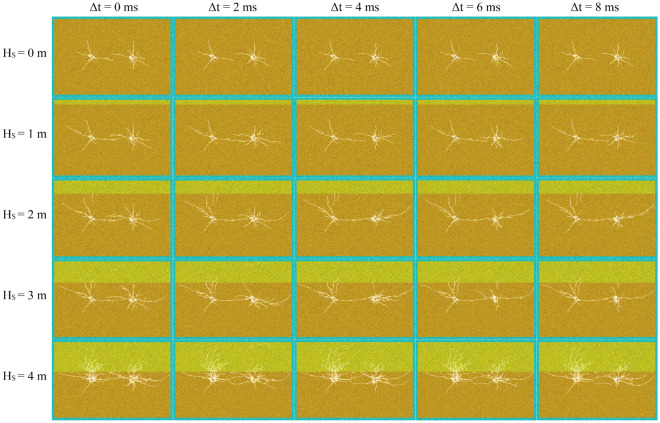
Initiation in hard rock.

**Figure 12:**
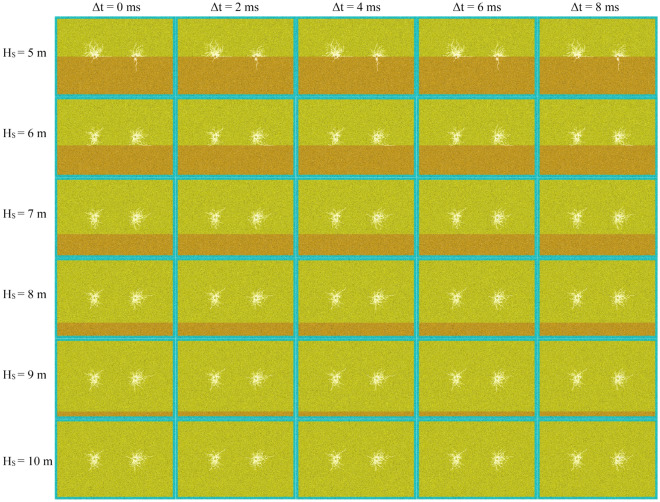
Initiation in soft rock.

According initiation position, experimental results can be divided into two parts, namely, initiation in hard rock (Fig. 11) and detonation in interface and soft rock (Fig. 12).

In the process of simultaneous or delayed initiation of two blastholes, the first blasthole produces the stress wave first. At this time, it is basically not affected by the second blasthole’s stress wave. When stress wave, generated by the second blasthole, reaches rock mass around the first blasthole, the main crack development process of the former basically ends. The stress wave of the former has propagated around the second blasthole when it is detonated. At this time, the initiation of the latter will be affected by the stress wave of the former, and the degree of influence is related to the initiation time. From the crack development state, the crack effect produced by the first initiation hole is less affected by the initiation time. And the main crack produced by the later initiation hole will be close to or even connected with the main crack produced by the first initiation.

#### Initiation in hard rock

1. Simultaneous initiation

When detonated simultaneously of two blastholes (Δ*t* = 0 ms) in hard rock (Fig. 11), the cracks in the connecting direction of the two blastholes will propagate to each other. And when the cracks are close to each other, they will avoid each other and form a “hook” or “butterfly ring”^6,40,41^. This phenomenon is more obvious when between interface and blasthole distance (it is called the distance for short) is larger than about twice crushing zone radius (the radius of crushing zone is about 0.5 m in 5 MPa confining pressure). As the distance decreases, the more stress waves it reflects, the more obvious the phenomenon of “hook”. So that the more overlapping parts of the main cracks in connecting line direction of blastholes. In addition, other main cracks also develop further with the continuous shortening of the distance. When two blastholes are detonated at the same time and the distance is less than about twice crushing zone radius, rock damage degree is serious, and the phenomenon of hooking is not obvious. It proves that the interface is close to blasthole, and reflecting ability for the stress wave is strong. Additionally, the existing cracks also hinder stress wave propagation. So that the stress wave between interface and blasthole converges more, and ultimately this phenomenon occurs.

(B) Delayed initiation

When delayed initiation (Δ*t* = 2, 4, 6, 8 ms) of two blastholes in hard rock (Fig. 11), the cracks in the connecting direction of the two blastholes will not appear obvious “hook”. Especially when the distance is larger than about twice crushing zone radius, “hook” cracks will gradually weaken or even disappear with increasing delay time.

When rock mass is pure hard rock (H_S_ = 0 m), the final effect of cracks is basically the same under different delay time, showing a trend close to the main crack. When H_S_ is 1 m, it also shows the trend of approaching the main cracks under different delay time, but the degree of approaching is also different with different delay time. With the increase of delay time, the main cracks generated by the later initiation hole will be closer to the main cracks generated by the first initiation. In addition, compared with the case of pure hard rock, when H_S_ = 1 m, the main cracks in the horizontal direction of the two blastholes further extend to both sides due to reflection effect of structural plane on stress wave. The most obvious influence of delayed initiation is that H_S_ = 2–3 m. When H_S_ is 2 m, crack effect caused by post initiation is obviously different with increasing delay time. When delay time is 2 ms, main cracks generated by the first initiation extend further to the direction of the blasthole, and the "hook" phenomenon becomes not obvious. When the delayed initiation time is more than 2 ms, the main cracks generated by the first initiation has been completely connected to the post initiation hole. When H_S_ = 2 m, from 0 to 8 ms, as increasing delay time, cracks parallel to the connection direction of the post initiation blasthole will further develop along the connection direction, while the cracks perpendicular to the connection direction will be restrained. The law when H_S_ = 3 m is basically the same as that when H_S_ = 2 m, so it will not be repeated.

When two blastholes are detonated in delay condition and the distance is less than twice crushing zone radius. At this time, due to reduction of the distance, rock mass damage between interface and blasthole is high, so impact of blasting with different delay time is not obvious.

In addition, from cracks distribution around the two blastholes, when detonating in hard rock and interface exists in hard rock, regardless of the delay time, rock mass damage degree around the first blasthole or crack extension length is more serious or wider than that of the later blast.

#### Initiation in soft rock

For initiation at the structural plane (Fig. 12), there are few cracks below the interface, and the range of cracks extension is also little. There are many cracks in soft rock mass on the upper side of interface, and the range of cracks extension is also large. It displays that soft rock is easy to crack due to its low strength. This conclusion can also be drawn from the crack distribution of initiation in soft rock. Due to the long distance between blastholes and the rapid attenuation of stress wave, interaction between blastholes is small, which is basically consistent with the final result of delayed initiation.

### Micro crack analysis

**Figure 13:**
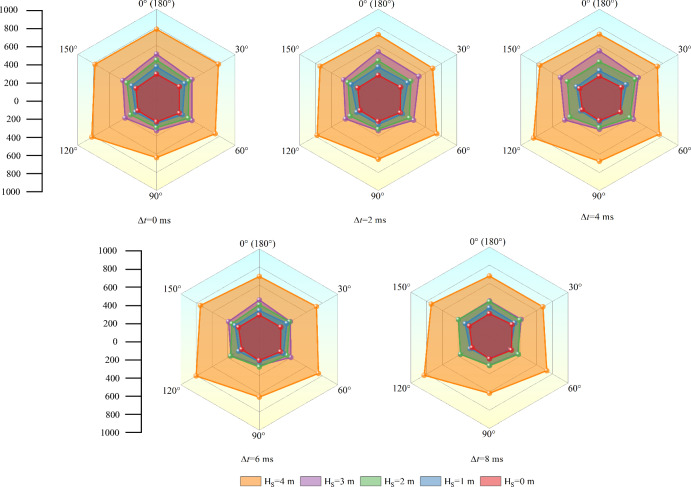
Micro crack information under hard rock initiation.

**Figure 14:**
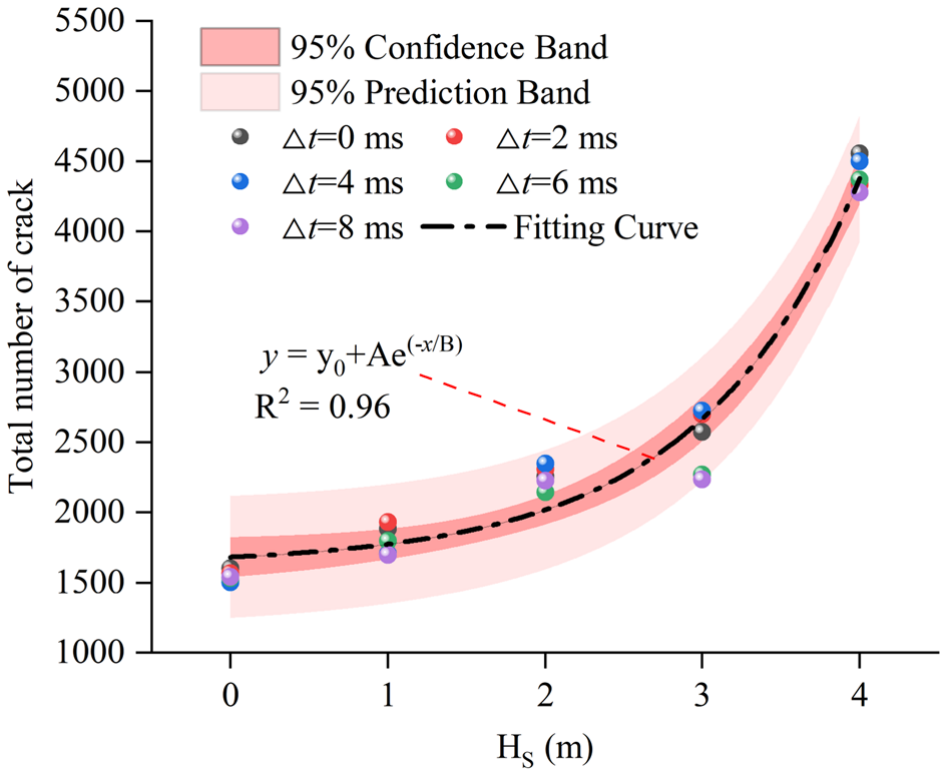
Statistics of total cracks.

Since cracks are chiefly concentrated near the blasthole, statistical angle and crack number reflect rock mass damage degree. When initiation in hard rock, crack information is counted, which is shown in crack rose diagram of PFC^2D^ under each working case, including crack angle and crack number. The results are shown in Figs. 13 and 14.

#### Initiation in hard rock

At different delay initiation time (Δ*t*), influence of soft rock thickness (H_S_) on micro cracks is basically the same. Under the condition of pure hard rock sample, overall distribution of crack number at each angle is relatively uniform. Due to interaction between the two blastholes, the number of cracks at a certain angle change slightly. It can be seen from Fig. 13 that under the case of pure hard rock, crack angle of about 90° is smaller than other angles. It can reflect that the crack in the direction of connecting with the blasthole is more developed than that in the direction perpendicular to the blasthole. And it can indicate that crack in the direction of connecting with the blasthole will be further developed in the direction of parallel blasthole regardless of simultaneous initiation or delayed initiation. Additionally, when Δ*t* is changeless, crack number in all directions will grow with increasing H_S_. It can indicate that the reflection of structural plane can promote the development of cracks in all directions around the blasthole, especially in the process of H_S_ growing from 3 to 4 m. However, as shown in Fig. 13, it does not increase uniformly in all directions. It shows that the change of crack is complex under the interaction between blastholes and the reflected stress wave of structural plane.

When the rock mass is pure hard rock (H_S_ = 0 m), crack number in all angles is basically unchanged under different delay times. Combined with the crack trend analyzed above, the delay time will change the direction of cracks, but there is no substantial change in the number of cracks, that is, it hardly influences rock mass failure degree. When H_S_ is constant, with the increase of Δ*t*, except under the working case of pure hard rock, the curve gradually flattens. That is, the cracks in the directions of 0° (180°) and 90° show a decreasing trend as a whole. It can be seen from the crack diagram in Fig. 11 that when the structural plane exists, the decrease of cracks about 0° (180°) is consistent with the weakening or even disappearance of "hook". While the decrease of cracks about the angle of 90° is consistent with the decrease of cracks in vertical direction of the later initiation blasthole. It shows that delay time will attenuate the number of cracks in the connecting direction of the blasthole and the vertical direction of the post initiation blasthole. That is, the delay initiation will weaken rock damage degree.

As shown in Fig. 13, the number of cracks varies greatly under various working cases. Therefore, this paper makes statistics on gross final cracks under cases of hard rock initiation, and the results are shown in Fig. 14. The total number of cracks under each working condition corresponds to the specific scatter points in Fig. 14. It can be seen from the scatter point diagram that H_S_ seriously influences the degree of rock failure, and the delay time will slightly change rock failure degree.

In order to research rock damage degree after blasting under cases of detonation in hard rock, the scatter diagram is fitted in this paper, and the fitting curve is shown in formula (7). According to the fitting results, when blasting in hard rock, rock damage degree grows exponentially with increasing H_S_.

10 $$y = {\text{y}}_{0} + {\text{Ae}}^{{( - x/{\text{B}})}}$$

where y_0_ is 1626.34, A is 56.12 and B is − 1.03.

#### Initiation in soft rock

**Figure 15:**
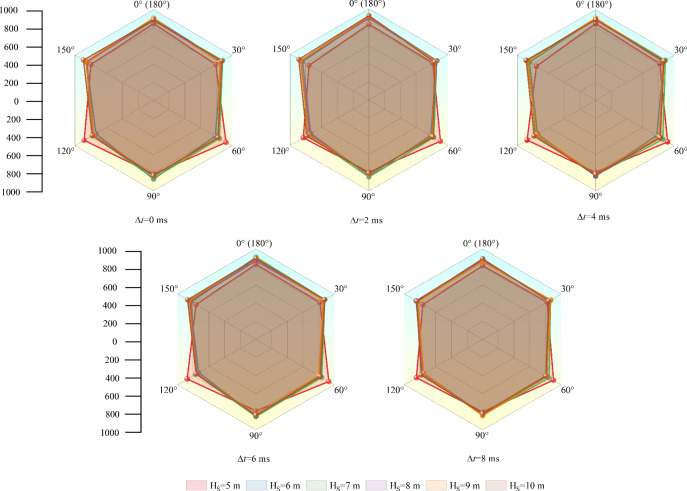
Micro crack information under soft rock initiation.

The crack information after initiation in structural plane and soft rock is shown in Fig. 15. After initiation in soft rock, crack number in all directions is basically the same, and hardly change with increasing H_S_. In addition, delay initiation time (Δ*t*) has little effect on the number of cracks. In all cases of H_S_ and Δ*t*, crack number in all directions is about 450. It can be seen that when detonation position in soft rock, the changes of H_S_ and Δ*t* hardly have influence on micro cracks. Micro information as well as variation rules of crack initiation in structural plane are basically consistent with that in soft rock. The gross crack number after initiation in soft rock as well as structural plane is almost unchanged under different delay time and soft rock thickness. Therefore, this paper does not make statistics on the number of cracks initiated in structural plane and soft rock.

### Analysis of rock fragment area

**Figure 16:**
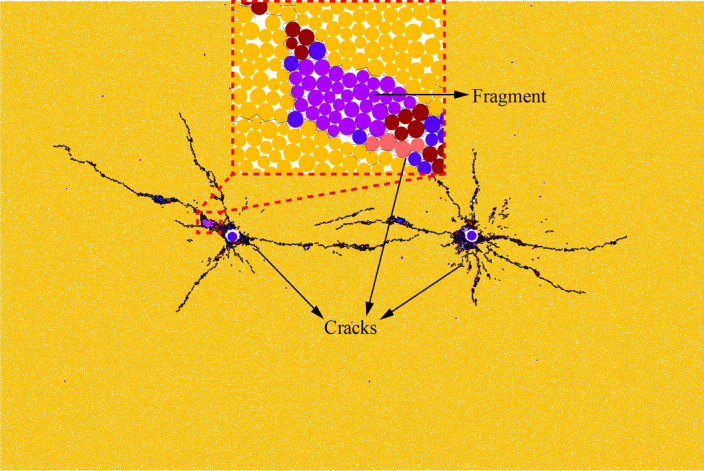
Relationship between fragments and cracks.

**Figure 17:**
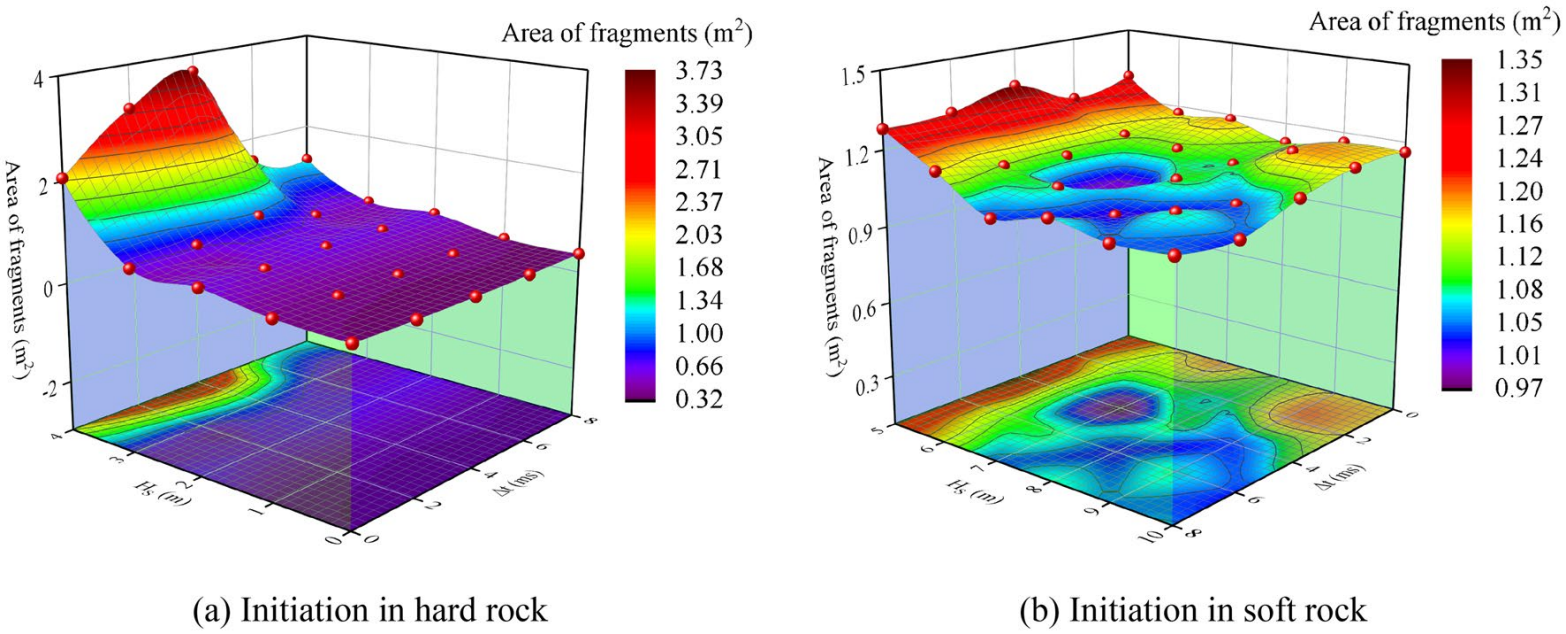
Fragments area statistics.

So as to research rock fragmentation after detonation, further statistics are made on the area of fragments under each case. As shown in Fig. 16, if the crack generated by blasting surrounds a group of particles, the surrounded group of particles is regarded as a fragment, that is, the purple part in Fig. 16. The statistical results are displayed in Fig. 17.

As shown in Fig. 17a, when initiating in hard rock, fragment area grows monotonically with increasing H_S_ at the same delay time. When H_S_ = 0, 1 and 2 m, the crushing zone is less affected by Δ*t*, and the variation is steady in the same H_S_ but different Δ*t*. When H_S_ = 3 and 4 m, the crushing zone is greatly affected by Δ*t*. Under the same H_S_, fragment area grows and then decays with increasing Δ*t*. When Δ*t* is 4 ms, fragment area reaches the maximum which is about 3.73 m^2^. When detonating in soft rock (Fig. 17b), the fragment area under various working cases is basically the same and stable at about 1 m^2^.

According to the conclusion obtained from Fig. 17 and the above crack network diagram, the initiation in hard rock has a larger range of cracks extension than that in structural plane and soft rock, but there are not many fragments formed. In addition, the fragment area value of hard rock initiation under various working cases (except H_S_ = 4 m) is lower than that of initiation in structural plane and soft rock. It suggests that initiation in both soft rock and structural plane are more conducive to the formation of fragments. Although the extension range of cracks in hard rock is relatively large, the fragmentation degree of rock mass is not as good as initiation in structural plane and soft rock.

**Figure 18:**
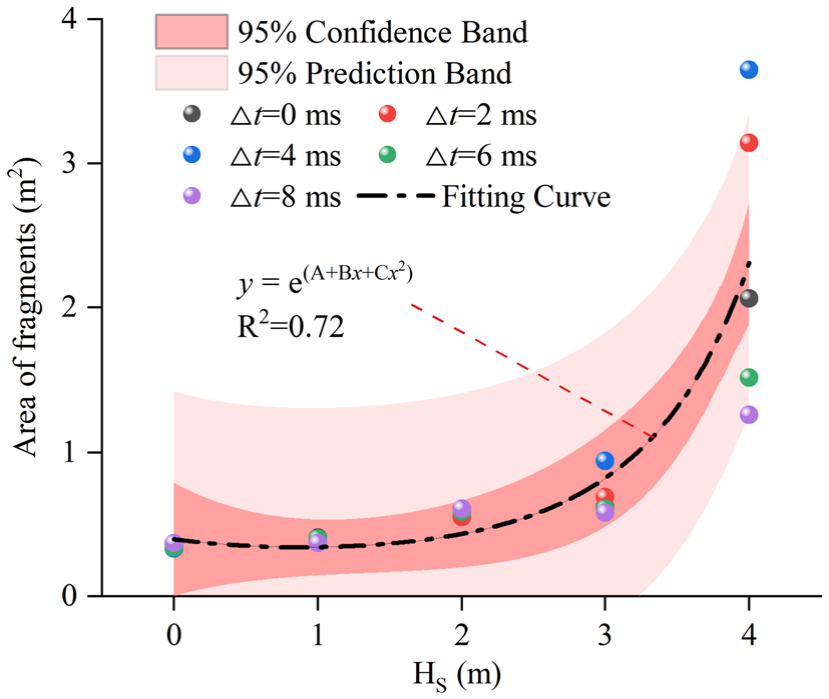
Change of fragments area.

Figure 18 shows the relationship between the fragments area and H_S_ and Δ*t* during detonation in hard rock. With increasing H_S_, fragment area increases exponentially. When H_S_ = 4 m, the fragments area reaches the maximum. Delayed initiation increases the fluctuation effect of fragments area value. With the increase of H_S_, the fluctuation effect caused by delayed initiation begins to increase, resulting in more discrete fragments area value. The fluctuation effect of delayed initiation is more obvious when H_S_ = 3 and 4 m. With increasing delay time, fragment area grows and then decays. When Δ*t* is 4 ms, fragment area arrives the maximum value. In addition, fragments area value is fitted in this paper. The fitting curve is shown in formula (8). Through the fitting curve, the variation trend of fragments area value with H_S_ and Δ*t* can be seen more clearly.

11 $$y{\text{ = e}}^{{{\text{(A + B}}x{\text{ + C}}x^{{2}} {)}}}$$

where A is − 0.93, B is − 0.35 and C is 0.20.

## Discussion

From mentioned analysis angles: crack network state, crack quantity and rock fragments, the delayed initiation time and between blasthole and interface distance greatly affect double-hole blasting effect. Therefore, to a certain extent, blasting effect will be more controllable if delayed time and initiation position are adjusted.

(1) From crack state, the crack initiation in hard rock extends longer than that in interface and soft rock, and main cracks are more developed. When the distance within 4–6 times crushing zone radius and detonates in hard rock, crack penetration in the connection direction of the blasthole is the best, and the main crack develops longer. When two blastholes are detonated simultaneously in hard rock, the main crack between blastholes will appear obvious “hook” phenomenon, which is not conducive to the formation of through crack. When the initiation time is delayed, the “hook” phenomenon can be significantly weakened by adjusting the initiation time of the two blastholes, so as to enhance the penetration effect of the crack.

(2) When detonation in hard rock, cracks extension range is large, but less fragments are formed. When interface is about twice crushing zone radius, fragments area is relatively large, which grows exponentially with increasing H_S_, and exponential function is fitted. However, when blasting in interface and soft rock, the range of cracks extension is less and the area of fragments is larger.

(3) It is generally believed that bedding, interface of the rock mass, is the stratification characteristic inside the sedimentary rock layer, and the rocks on both sides of the bedding are different in rock property. The joint is the fracture surface of the rock mass, and the rock properties on both sides are similar. Therefore, due to the different definitions of these two structural planes, this paper does not choose the method which some scholars adopted to calibrate the joints, but adopts the method of 'screening'. In the process of parameter assignment of natural rock mass, the whole rock mass is given hard rock parameters first, and then the parameters of some rock mass are replaced by soft rock parameters after screening.

(4) This paper is to carry out double-hole blasting experiments based on soft-hard composite rock strata considering delay initiation effect, and to study the influence of the distance on crack propagation characteristics. Compared with the research results of previous scholars, the innovation is mainly considering the special geological conditions of soft-hard composite rock strata. However, experiments, in this paper, are carried out under the condition of 5 MPa in-situ stress. Variation of in-situ stress and more complex geological conditions will be another major obstacle to the controlled blasting effect. Therefore, it is necessary to carry out research on these issues.

## Conclusions

By using Particle Flow Code (PFC^2D^), numerical simulation verification is carried out in this paper. Under the condition of reasonable verification results, considering delay initiation time and soft-hard rock strata, double-hole blasting experiments are conducted. Then, the result is analyzed from several angles, including crack state, crack quantity and rock fragments. The conclusions are as follows:

1. When between interface and blasthole distance is larger than about twice crushing zone radius and detonates in hard rock, the smaller the distance, the more obvious the “hook” phenomenon caused by the main cracks of the two blastholes. The delayed initiation will weaken the “hook” phenomenon and even make it disappear.
2. If detonation in pure hard rock, due to interaction between blastholes, cracks in the connection direction of blastholes will be further developed. The delay time will change the direction of cracks, but there is no substantial change in rock mass damage degree. When detonation in hard rock and existing the interface, compared with simultaneous blasting, delayed blasting will weaken rock mass damage degree around the later blasthole, and the weakening effect will grow with increasing delay time. According to crack information, detonation in hard rock, the formula of crack number varying with soft rock thickness is fitted.
3. When detonating in hard rock, the range of cracks extension is large, but less fragments are formed. The law is opposite to that in structural plane and soft rock. The fragments area grows exponentially with increasing H_S_, and the exponential function is fitted.

## Data Availability

All data generated or analysed during this study are included in this published article.
